# Correlation between Tribological Properties and the Quantified Structural Changes of Lysozyme on Poly (2-hydroxyethyl methacrylate) Contact Lens

**DOI:** 10.3390/polym12081639

**Published:** 2020-07-23

**Authors:** You-Cheng Chang, Chen-Ying Su, Chia-Hua Chang, Hsu-Wei Fang, Yang Wei

**Affiliations:** 1 Department of Chemical Engineering and Biotechnology, National Taipei University of Technology, 1, Sec. 3, Zhongxiao E. Rd., Taipei 10608, Taiwan; youchengchang@gmail.com (Y.-C.C.); chenying.su@gmail.com (C.-Y.S.); t104320088@ntut.org.tw (C.-H.C.); 2 Institute of Biomedical Engineering and Nanomedicine, National Health Research Institutes, No. 35, Keyan Road, Zhunan Town, Miaoli County 35053, Taiwan

**Keywords:** poly (2-hydroxyethyl methacrylate), bio-friction, lysozyme adsorption, contact lens-related discomfort, protein–protein effects, conformational changes

## Abstract

The ocular discomfort is the leading cause of contact lens wear discontinuation. Although the tear proteins as a lubricant might improve contact lens adaptation, some in vitro studies suggested that the amount of adsorbed proteins could not simply explain the lubricating performance of adsorbed proteins. The purpose of this study was to quantify the structural changes and corresponding ocular lubricating properties of adsorbed protein on a conventional contact lens material, poly (2-hydroxyethyl methacrylate) (pHEMA). The adsorption behaviors of lysozyme on pHEMA were determined by the combined effects of protein–surface and protein–protein interactions. Lysozyme, the most abundant protein in tear, was first adsorbed onto the pHEMA surface under widely varying protein solution concentrations to saturate the surface, with the areal density of the adsorbed protein presenting different protein–protein effects within the layer. These values were correlated with the measured secondary structures, and corresponding friction coefficient of the adsorbed and protein covered lens surface, respectively. The decreased friction coefficient value was an indicator of the lubricated surfaces with improved adaptation. Our results indicate that the protein–protein effects help stabilize the structure of adsorbed lysozyme on pHEMA with the raised friction coefficient measured critical for the innovation of contact lens material designs with improved adaptation.

## 1. Introduction

The use of contact lenses has become increasingly popular for vision correction and cosmetic reasons over prescribed spectacles [1,2]. However, the insertion of a contact lens into the eye does change the situation at the ocular surface [3] and often causes the wearer contact lens-related discomfort (CLD). CLD is a condition characterized by adverse sensation [4,5] resulting from reduced compatibility between the contact lens and the ocular environment [6], which usually leads to discontinuation of contact lens wear [7]. Various factors that may be related to CLD include lens material designs [8,9] and ocular changes such as varied tear composition and external variations, which include the use of medications, room humidity, or air temperature [10]. Therefore, the management of CLD remains a challenge, and research into CLD aims to determine which factor will improve contact lens adaptation.

A more recent area of interest is lubricity and friction involved during contact lens wear, with current studies suggesting that the reduced friction between the cornea, lens surface, and lid margin is the key to lens comfort [6]. Meanwhile, proteins have been shown to act as aqueous boundary lubricants in physiology, with adsorbed synovial fluid proteins in articular joints [11] and saliva proteins in the oral cavity providing a low-shear-strength fluid film that lubricates the system [12]. Similarly, tear proteins might be able to provide aqueous boundary lubrication as well.

Immediately after being placed on the eye, the contact lenses are subject to rapid deposition of the surrounding biomolecules, including proteins, lipids, mucins electrolytes, and water in the tear film [13]. Of these, the tear film consists of more than 400 different proteins, ranging in size from 10 to 2360 kDa [14], and lysozyme is the most abundant in tears. The average pH of the tear film is 7.4, which results in lysozyme (14.3 kDa, pI = 11.4) being positively charged with a substantial degree of adsorption to negatively charged substrates [14]. The presence of the adsorbed proteins on a contact lens is significantly related to CLD depending on different lens materials applied [15,16,17,18]. However, some in vitro studies further suggested that the lubricating performance of adsorbed proteins could not be simply explained by the amount of adsorbed lysozymes observed [11,14,17,19,20]. Adsorption induced structural changes of the tear proteins on the contact lens may play an essential role in the rise or reduced feelings of discomfort due to their lubricating properties involved but are rarely subjected to a similar discussion, probably due to the complex mechanisms governing protein unfolding behaviors.

Proteins in the tear film are present in native structures. Still, when proteins adsorb onto a contact lens surface, the forces between the proteins, adsorbent surface, and environmental molecules may alter the thermodynamic state of the system leading to inevitable shifts in an adsorbed protein’s structure [14,15,21]. The extent to which unfolding will occur is primarily determined by the combined effects between protein–protein, protein–surface, and internal stability of the protein [22]. Protein–protein interactions could be defined as interactions between neighbor proteins, usually present as the sterical block of further unfolding and spreading of an adsorbed protein when other adsorbed ones occupy its adjacent area surfaces, in which case, the protein solution concentration that will influence the filling rate of neighbor proteins could be related to the degree of protein–protein effects involved [23].

This study thus aims to investigate the association between subjective ocular CLD and conformational changes of adsorbed tear proteins on contact lens surfaces with protein–protein effects involved. Lysozyme was chosen as the model protein and adsorbed from varying protein solution concentrations to have different protein areal density on the adsorbent surface from which the varied PPI effects within the adsorbed lysozyme layer could be observed on a poly-2-hydroxyethyl methacrylate (pHEMA)-based lens surface. The pHEMA is a conventional material for contact lenses due to its excellent biocompatibility and is categorized by the Food and Drug Administration (FDA) as a Group I (nonionic, <50% water) material [14]. Following this, the conformational structure of adsorbed lysozymes was quantified using circular dichroism (CD). Finally, the measurement of the friction coefficient was conducted using an in vitro model to simulate the bio-friction generated in the eyes [24,25] to quantify the lubricating performances of the same protein–surface system. The coefficient of friction is defined as the ratio of friction force measured to the force applied from the normal direction, with the friction coefficient and friction force reduced by introducing a lubricant film between two solid surfaces at a constant load [26]. Based on the fundamental understandings regarding the adsorbed tear proteins and corresponding lubricating performance, our results may provide useful references for developing new materials of contact lenses or contact lens care solutions, to reduce discomfort for contact lens wearers.

## 2. Materials and Methods

### 2.1. Contact Lens and Reagents

The pHEMA-based contact lenses used in this study were Hydron Eye Secret Aspheric Daily Contact Lenses (Polymacon, Yung Sheng Optical Co., Ltd., Taichung, Taiwan). Lysozyme (Sigma-Aldrich, St. Louis, MO, USA) in physiological saline (pH 7.0–7.2) was prepared in this study. The lysozyme concentration was about 1.9 mg/mL in human tears film [27,28].

### 2.2. Method for Protein Adsorption on Contact Lenses

Each contact lens was taken out of the packaging and the excess liquid was removed before using it. The contact lenses were immersed in 30 mL lysozyme solutions of different concentrations (0.5, 0.7, 1.0, 1.5, and 1.9 mg/mL) for two hours. After this, the protein solution was changed to pure physiological saline (pH 7.0–7.2) for another two hours for desorption of loosely adsorbed lysozyme on surfaces [22].

### 2.3. Preparation of pHEMA Surfaces

Customized glass slides (0.95 × 4.00 × 0.15 cm^3^) were used to fit the CD cuvettes. Poly (2-hydroxyethyl methacrylate) (pHEMA, Sigma) surfaces were spin-coated onto glass slides from 2%(*w*/*w*) pHEMA solution at 2000 rpm for 15 s in 0.1 mL methanol and incubated at 37 °C for 10 min.

The glass substrates used for the spin-coating process were cleaned by sonication in piranha containing (3:1 (*v*/*v*) 95–98% sulfuric acid (H_2_SO_4_, Scharlau, Barcelona, Spain)/hydrogen peroxide aqueous solution 30% (H_2_O_2_, SHOWA, Saitama, Japan)) and basic solution (1:1:5 (*v*/*v*/*v*) ammonia solution (NH_4_OH, SHOWA)/H_2_O_2_/H_2_O). After cleaning the glass with pure water, the glass was left at 37 °C until it was thoroughly dried [22].

### 2.4. Analysis of the pHEMA Surface

The surface element was analyzed with X-ray photoelectron spectroscopy (XPS, VG ESCALAB250, VG Scientific, East Grinstead, UK). The solid sample was prepared and placed under a ultra-high vacuum (<9 × 10^−8^ mBar). The focused X-ray energy was adjusted to 1.5 keV, and the z-axis position was confirmed to focus on the object’s surface. The element spectrum was clicked, and the orbit was set. The number of scans was five times each time, and the energy was 1 electron volt (eV). The corresponding raw data are shown in Section S1 of the Supplementary Materials.

The contact angle of the surface was tested with a CA-D type contact angle meter (100 SB Sindatek, Taiwan). The deionized water droplets were placed on the surface of the object, and the angle was measured.

The surface roughness was analyzed with atomic force microscopy (AFM, XE-100, Park System, Ilsan, Korea). The sample surface was required to be flat and attached to the stage, with a scan range of 10 μm × 10 μm. The detailed descriptions are given in Section S2 of the Supplementary Materials.

### 2.5. Protein Adsorption Areal Density Measurement

The protein concentrations were verified via circular dichroism spectroscopy (CD, Spectropolarimeter J-810, Tokyo, Japan). Different protein solutions were prepared using physiological saline (pH 7.0–7.2) as a solvent, including 0.01 mg/mL, 0.02 mg/mL, 0.03 mg/mL, 0.04 mg/mL, and 0.05 mg/mL under room temperature and measured at a wavelength (λ) of 205 nm [29]. The measured absorbance values vs. protein concentrations calibrated by a BCA assay [30] were noted to construct the standard calibration curve.

The molar extinction coefficient of the protein (*ε*_205_) in solution at 205 nm was then determined by recording the background-corrected absorbance at different solution concentrations, as mentioned in Section 2.2, to determine the areal density of the adsorbed protein. Subsequently, the molar extinction coefficient of lysozyme in the saline was obtained from the slope of the absorbance (A_205_) vs. (Csoln × L) plot. Thus, the areal density of the adsorbed lysozyme was determined by the following equation [31]:

(1) $$Q_{ads}=\frac{A_{205}}{\varepsilon_{205}}$$

where A_250_ is the background corrected absorbance at 205 nm, and *ε*_205_ is the molar coefficient that was determined for the protein solution at a wavelength of 205 nm.

### 2.6. Measurement of the Structural Changes of Lysozyme Adsorbed on Soft Contact Lens

The native structure of proteins in physiological saline and the following adsorption-induced secondary structure changes of these adsorbed proteins were determined using circular dichroism (CD). In this study, the CD was operated at room temperature over the wavelength range between *λ*_1_ = 280 nm and *λ*_2_ = 190 nm. The path length was 1 cm, and the scan speed was 50 nm/min in solution status and 100nm/min in adsorbed protein solution, respectively, with standard (100 mdeg) sensitivity. Each spectrum was averaged three times.

The secondary structure in solution and of adsorbed protein was estimated by converting the background-corrected CD signals to molar ellipticity (*θ_mol_*) using the following equations [32]:

(2) $$\theta_{mol}=\frac{\theta_{raw}\times M}{1000\times \theta_{soln}\times L}$$

(3) $$\theta_{mol}=\frac{\theta_{raw}\times M}{10000\times Q_{ads}}$$

where *θ_raw_* is the background corrected raw CD signal, *L* is the path length of the cuvette (cm), *C_soln_* is the solution concentration of the protein (g/mL), *Q_ads_* is the surface density of adsorbed protein(g/cm^2^), and *M* is the mean residue molecular weight of 112 g/mol.

From the obtained molar ellipticity (*θ_mol_*), the secondary structure content was estimated by DichroWeb, an online database [33,34].

### 2.7. In Vitro Testing System of Friction Coefficient

*In vitro* contact lens friction tests were measured with a CETR universal micro-tribometer-2 (UMT-2, Bruker, Campbell, CA, USA) using the procedure previously described by Su et al. to simulate the ocular environment [24]. A contact lens adsorbed with lysozyme was fixed on the top stage while the glass was used as the bottom. The contact lens was then rubbed against the quartz glass as the intraocular or natural crystalline lens in the presence of 10 mL of physiological saline [35,36]. A previous study suggested that the eyelid force exhibited towards the eye was in the range of 47–149 milli-Newtons (mN) [37]. The normal force applied in this study was 60 mN due to its most stabilized noise to signal ratio with the rotation speed of 1 rpm. The rotation radius was 10 mm with 5 mm of radius contact area, and the rotation time was 900 s. The quartz glass was cleaned with 75% ethanol after each friction test, and each condition was repeated more than three times.

### 2.8. Statistical Analysis

Differences in friction coefficients and lysosomal structure changes were assessed by Student’s *t*-test to make an allowance of comparisons. A value of *p* < 0.05 was considered significant.

## 3. Results

### 3.1. Characteristics of pHEMA Surfaces

The surface contact angle was measured as 41.2 ± 0.5 (average ± standard deviation, *n* = 3), similar to the reported values [38,39]. Elemental composition was measured by X-ray photoelectron spectroscopy with the analysis spectrum mentioned in Supplementary Materials Figure S1. Varied surface roughness based on different coating parameters were collected from AFM images and are shown in Supplementary Materials Figure S2. All of the measured values reported fell within the expected range [40].

### 3.2. The Areal Density of Adsorbed Proteins and Protein–Protein Effects

**Figure 1:**
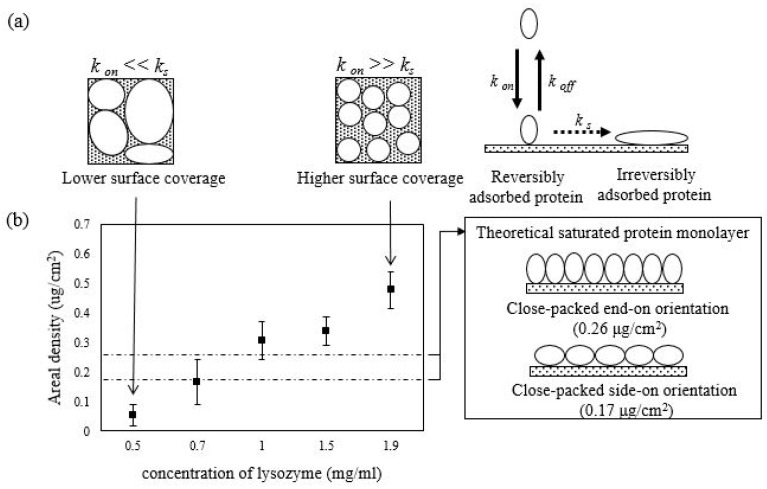
(**a**) The interaction of a protein with the surface may occur as a dynamic exchange process with adsorption rate and desorption rate represented by *k*_on_ and *k_off_*,, respectively. The adsorbed proteins may undergo surface-induced unfolding and spread out over the surface to increase their footprint on the surface [41] with the unfolding rate expressed as *k*_s_. (**b**) The adsorption capacity represented by the areal density of the adsorbed lysozyme protein on the pHEMA surface was measured at different protein solution concentrations. The error bars denote the mean ± SD for *n* = 3.

Figure 1 presents a plot of the areal density of the adsorbed lysozyme on the pHEMA surface for each protein solution concentration. As shown, protein solutions resulted in distributed areal densities, with the amount of adsorbed protein from higher solution concentrations falling within the areal densities corresponding to the theoretical limits for a saturated surface. Theoretically, lysozyme could be organized in a close-packed side-on orientation (0.17 μg/cm^2^) and close-packed end-on orientation (0.26 μg/cm^2^) to saturate the surface with a monolayer [22].

### 3.3. The Protein–Protein Effects and Protein Secondary Structure

**Figure 2:**
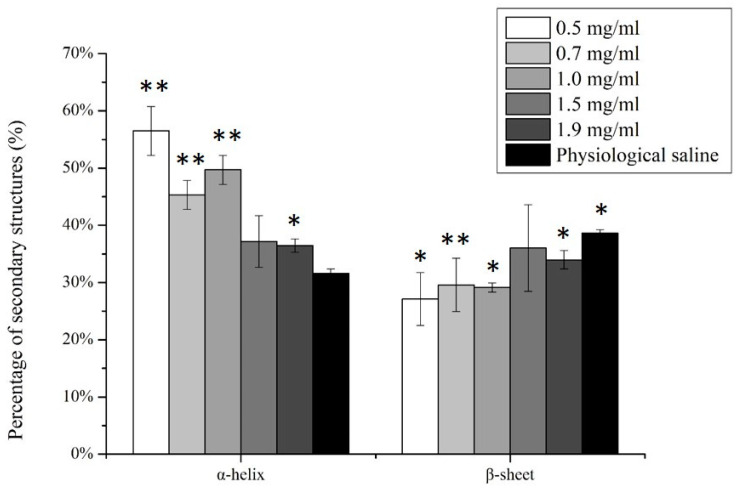
Secondary structural contents (%) of adsorbed lysozyme on pHEMA surface from different solution concentrations. The secondary structure of lysozyme measured in physiological saline solution was studied as the control. The error bars denote the mean ±SD for *n* = 3. *and ** represent *p* < 0.05 and *p* < 0.01, respectively.

The influence of protein–protein interactions on the secondary structure of adsorbed lysozyme is presented in Figure 2. As shown, secondary structures of lysozyme adsorbed from increased solution concentration were comparatively retained on the pHEMA surface. There was a significant reduction in the helical structure. In contrast, a substantial increase in percent sheet content was observed for the lysozyme layers adsorbed from solutions at lower protein concentration when compared to native protein structure in physiological saline.

### 3.4. The Structural Changes of Adsorbed Lysozyme and the Friction Coefficient of Lysozyme Covered pHEMA Contact Lens Surface

**Figure 3:**
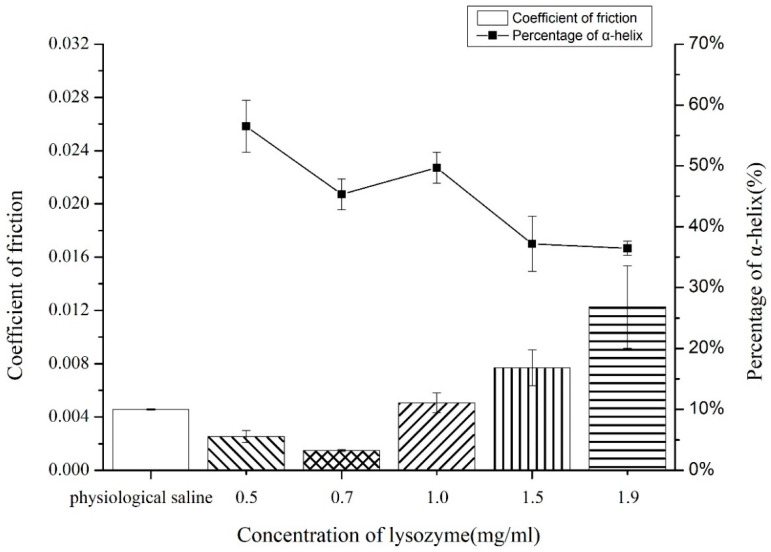
The friction coefficient of the Polymacon contact lens covered with lysozyme adsorbed from different solution concentrations. The friction coefficient of the Polymacon surface with no protein adsorbed in a physiological saline solution was measured as a control. The error bars denote the mean ± SD for *n* = 3.

Figure 3 presents a plot of the percent helical content of the adsorbed lysozyme on pHEMA-based contact lens (Polymacon) with corresponding friction coefficient values for each protein solution concentration. As shown, the coefficient of friction values was reduced when the lysozyme was adsorbed from the diluted protein solution concentrations, with the increased secondary structural changes as indicated by the increased helical content (%).

## 4. Discussion

The pHEMA is a biocompatible and water-absorbing material used to make contact lenses or medical delivery systems [42,43]. The presence of carbonyl (C=O) and one hydroxyl (–OH) groups on each side chain in pHEMA subsequently reduce the hydrophobicity of the surface [44].

As indicated in Figure 1b, the interaction of the lysozyme with the pHEMA occurs rapidly, with proteins adsorbed from different solution concentrations resulting in the widely distributed areal densities, as shown in Figure 1a. The initial interaction of a protein with a surface may occur as a dynamic exchange process with adsorption rate and desorption rate represented by *k*_on_ and *k_off_*, respectively. However, adsorbed proteins may undergo surface-induced unfolding and spread out over the surface to increase their footprint on the surface [41] with the unfolding rate expressed as *k*_s_, which is usually an irreversible process [20]. When protein is adsorbed from higher solution concentration, there is a subsequent increase in adsorption rate. When the adsorption rate is faster than the unfolding rate of an adsorbed protein on the surface (i.e., *k*_on_ >> *k*_s_), the degree of spreading of a protein over the surface will be inhibited by the neighboring adsorption sites being occupied by other adsorbing proteins. This condition is indicated by the significant protein–protein interactions with the saturated monolayer coverage of protein on the surface (i.e., minimized footprint). In this study, the areal densities of adsorbed lysozyme correspond to the theoretical limits for a saturated surface for a monolayer of protein organized in a close-packed side-on orientation (0.17 μg/cm^2^) and close-packed end-on orientation (0.26 μg/cm^2^) [22]. On the other hand, when protein is adsorbed from diluted solution concentrations, there is a relatively higher unfolding rate when compared to the adsorption rate (i.e., *k*_on_ << *k*_s_), in which case the proteins on the surface tend to spread out and reach their maximum footprint depending on the protein–surface interactions involved before the neighboring sites being occupied, with a relatively lower amount of protein being adsorbed, which presents the minimized protein–protein interactions [23]. The broad range of areal density observed in Figure 1, which could be controlled by varying the protein solution concentrations from which the protein is adsorbed, thus could be proportionally correlated with a broad distribution of protein–protein interactions involved. For example, when adsorbed from the diluted solution concentration, there will be a lower areal density of adsorbed proteins with subsequent weaker protein–protein interactions.

The data from Figure 2 further present a much clearer correlation between the protein–protein interactions and the stability of adsorbed lysozyme on a pHEMA surface. The increased protein–protein interactions were represented by the higher real density of adsorbed proteins, from which it is clearly shown that protein–protein interactions tend to stabilize the structure of adsorbed lysozyme on pHEMA. Little structural changes were observed when compared to the native lysozyme structure in physiological saline. For example, the highest areal density (i.e., representing the most significant protein–protein interactions involved) was observed when adsorbing the protein from a solution concentration of 1.9 mg/mL. There is only a 15% decrease in the native-state percent sheet (38% to 33%) and a 15% increase in the native-state percent helicity (30% to 35%), while at the lowest areal density observed when protein was adsorbed from 0.5 mg/mL solution concentrations, the protein–protein interaction was minimized.

The protein–surface interactions further induced the sheet and helical structures to decrease to only 28% (i.e., 25% loss in percent sheet) and increase to 58% (i.e., more than 90% increase in percent helicity), respectively.

Based on these results observed, the pHEMA surface with a large density of hydrogen bondable groups may interact with lysozyme by competing with the hydrogen bonds that stabilize the protein secondary structures on the surface [11,45], from which the lysozyme structure is destabilized due to the protein–surface interactions involved. On the other hand, the protein–protein interactions tend to inhibit the unfolding of adsorbed lysozyme from neighbor proteins, thus helping to stabilize the protein structures on a pHEMA surface.

**Figure 4:**
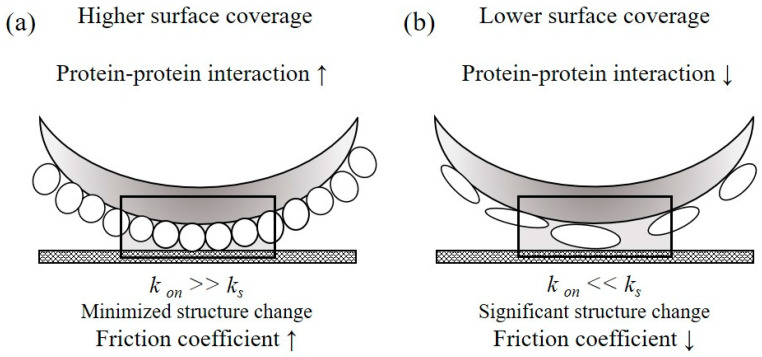
Influence of protein–protein effects on the surface friction coefficient values. A comparison of contact lens surfaces with different surface protein coverages causes separate boundary lubrication, resulting in schematic diagrams with varying friction coefficients. Under the minimized structure change (**a**), the high shear strength layer was observed at the interface. In the case of lower surface coverage (**b**), there is the opposite result. The adsorption rate of interacting the protein with the surface was represented by *k*_on_, and the unfolding rate of adsorbed protein on the adsorbent surface was expressed as *k*_s_.

Finally, an apparent correlation was observed between the coefficient of friction measured from the protein covered contact lens surface (Polymacon) and percent helical content within the adsorbed lysozyme layer. Polymacon is a pHEMA-based material with a nonionic hydrophilic 2-hydroxyethylmethacrylate (HEMA) cross-linked with ethylene glycol dimethacrylate [46]. Our results suggest that the friction coefficient is primarily being influenced by the structures of adsorbed proteins, with the friction coefficient reduced from the increased helical content from the native state structures, reflecting the reduced friction forces between two solid surfaces at a constant load, in which case, when compared to the control group (i.e., pure physiological saline at the interface), the lysozyme in the native structure was shown to be a poor boundary lubricant, which might be considered as a high-shear-strength layer at the interface. On the other hand, the lubricating properties of the unfolded lysozyme upon adsorption on a Polymacon surface might be attributed to the hydrophilic moieties exposed to the aqueous environment. Thus, the water molecules trapped at the surface might provide a lubricious, low-shear-strength, fluid film [11,12], as shown in Figure 4.

Therefore, when these data are compared with results from Figure 2, they further suggest that protein–protein interactions primarily influence the friction coefficient involved by preserving the structures of adsorbed lysozyme on a pHEMA, which in turn increases the friction coefficient observed. However, a complete study considering more complex model fluids, including the ionic effects, interactions between multiple proteins, and physiological temperature settings to present the biomolecules within the tear film, is required in our future study.

## 5. Conclusions

In this study, the relationship between the adsorption responses of lysozyme from varied protein solution concentrations and the friction between the surfaces of protein-covered contact lens material was studied at a molecular level. The low friction between two solid surfaces might be attributed to the fluidity of the hydrated and unfolded lysozyme layers from which the degree to which protein–protein interactions limit the unfolding of lysozyme on a pHEMA surface can thus be related to protein lubricating properties and be simply controlled by adjusting the concentration of the protein in solution, which influences the CLD of lens wear.

## Supplementary Materials

The following are available online at https://www.mdpi.com/2073-4360/12/8/1639/s1, Figure S1: X-ray photoelectron spectrometer of pHEMA used in this study. Figure S2: AFM images and surface roughness analysis for commercially available contact lenses and pHEMA surface made with different coating parameters.
