# Supplementary material for: Correlation between Tribological Properties and the Quantified Structural Changes of Lysozyme on Poly (2-hydroxyethyl methacrylate) Contact Lens
Source: Polymers (Basel). 2020 Jul 23;12(8):1639. doi: 10.3390/polym12081639 (PMC7464607; doi:10.3390/polym12081639)
Supplement: Supplementary file 1 [file polymers-12-01639-s001.pdf]

Correlation between tribological properties and the quantified structural changes of lysozyme on poly (2-hydroxyethyl methacrylate) contact lens

You-Cheng Chang[1], *Chen-Ying Su*[1], *Chia-Hua Chang*[1], *Hsu-Wei Fang*[1,2]*, *Yang Wei*[1*]

1Department of Chemical Engineering and Biotechnology, National Taipei University of Technology, 1, Sec. 3, Zhongxiao E. Rd., Taipei 10608, Taiwan
2Institute of Biomedical Engineering and Nanomedicine, National Health Research Institutes. No. 35, Keyan Road, Zhunan Town, Miaoli County 35053, Taiwan

Corresponding authors: Dr. Hsu-Wei Fang/ Dr. Yang Wei
Email addresses: hwfang@ntut.edu.tw/ wei38@ntut.edu.tw

This supporting information includes: (*i*) X-ray photoelectron spectrometer data of poly (2-hydroxyethyl methacrylate) surface. (*ii*) The surface roughness of commercially available contact lenses and pHEMA surface.

S.1 X-ray photoelectron spectrometer data of poly (2-hydroxyethyl methacrylate) surface

The surface element was analyzed with X-ray photoelectron spectroscopy (scientific ESCALAB 250, VG United Kingdom). The solid sample was prepared and placed under an ultra-high vacuum (<$9x10^{-8}$ mBar). The focused X-ray energy was adjusted to 1.5keV, and the z-axis position was confirmed to focus on the object's surface. The element spectrum was clicked, and the orbit was set. The number of scans was five times with the rise of 1 electron volt (eV) each time. It was evident that the appeared two peaks, namely at 285 and 533 eV from the measured data, corresponds to the intense carbon binding energy and oxygen binding energy, respectively.

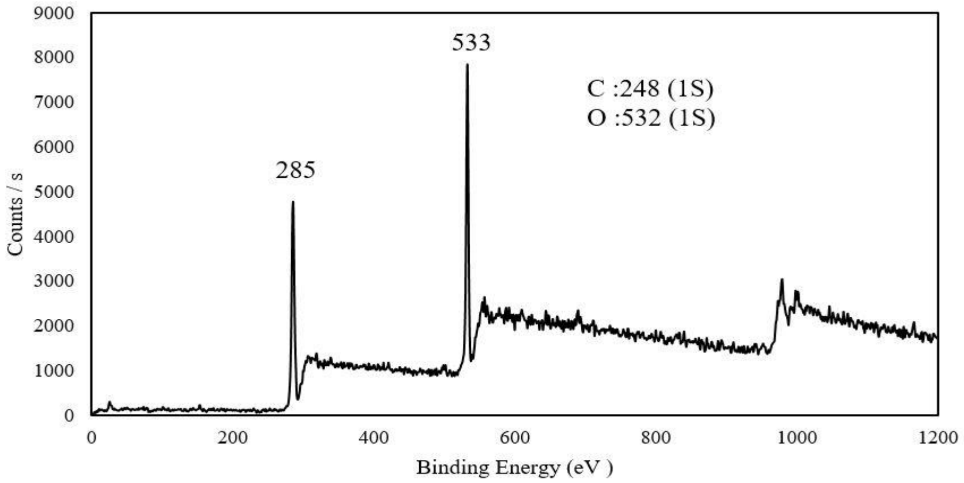

Figure S1. X-ray photoelectron spectrometer of pHEMA used in this study

S.2 Surface roughness of commercially available contact lenses and pHEMA surface.

The surface roughness was analyzed with atomic force microscopy (AFM, XE-100, Park). The micro-arm was used to sense and amplify the force between the sharp probe on the cantilever and the test sample atoms. The sample surface was required to be flat and attached to the stage, and the vacuum machine was turned on. The Z-axis was adjusted until the tip of the tip was clear. The probe frequency was set to the range between 0 and 1000 kilohertz (kHz), scan range: 10x10 μm, and z-axis range: <12 um. The pHEMA coating surfaces were made to mimic the commercial contact lens surface by adjusting the following three different spin coating parameters:

1. 2 % (w/w) pHEMA solution, 2000 rpm spin coating rate
2. 1 % (w/w) pHEMA solution, 2500 rpm spin coating rate
3. 1 % (w/w) pHEMA solution, 3000 rpm spin coating rate

The surface roughness of commercial contact lenses was 3.0 nm (Fig. S2(a)). The surface roughness of the pHEMA coating surface at an application rate of 2000 rpm was 3.6 nm (Fig. S2(b)), the roughness was 12.2 nm at a rate of 2500 rpm (Fig. S2(c)), and the roughness at a speed of 3000 rpm was 7.0 nm (Fig. S2(d)). From which the condition (2) mentioned in Fig. S2(b) was chosen with 2000 rpm to make the pHEMA coatings in this study for further experiments.

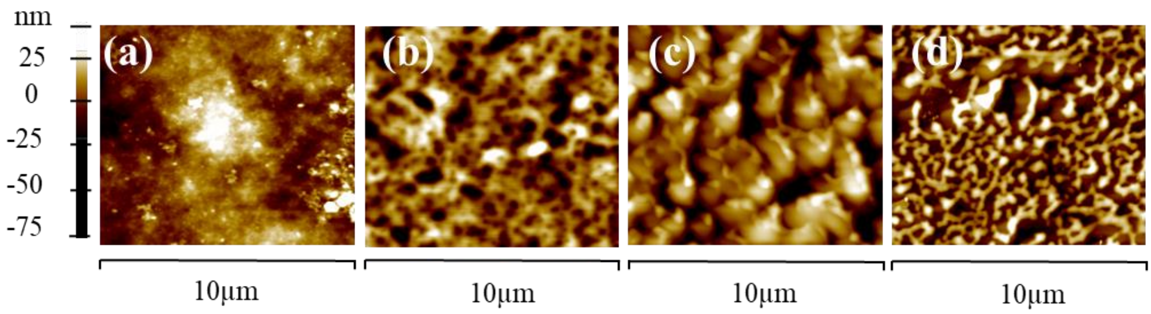

Figure S2. AFM images and surface roughness analysis for commercially available contact lenses and pHEMA surface made with different coating parameters. (a) Commercial contact lenses with Ra = 3.0 nm, (b) 2%(w/w) pHEMA made of 2000 rpm for 15 seconds with Ra = 3.6 nm, (c) 1%(w/w) pHEMA made of 2500 rpm for 15 seconds with Ra = 12.2 nm and (d) 1%(w/w) pHEMA made of 3000 rpm for 15 seconds with Ra = 7.0 nm.
